# Impact of Graphene Oxide on Zero Shear Viscosity, Fatigue Life and Low-Temperature Properties of Asphalt Binder

**DOI:** 10.3390/ma14113073

**Published:** 2021-06-04

**Authors:** Abbas Mukhtar Adnan, Chaofeng Lü, Xue Luo, Jinchang Wang

**Affiliations:** 1 College of Civil Engineering and Architecture, Zhejiang University, 866 Yuhangtang Road, Hangzhou 310058, China; abbasmadnan@zju.edu.cn (A.M.A.); xueluo@zju.edu.cn (X.L.); wjc501@zju.edu.cn (J.W.); 2 Soft Matter Research Center, Zhejiang University, Hangzhou 310027, China

**Keywords:** asphalt binder, graphene oxide, fatigue, zero shear viscosity, LAS test, low-temperature cracking

## Abstract

This study has investigated the impact of graphene oxide (GO) in enhancing the performance properties of an asphalt binder. The control asphalt binder (60/70 PEN) was blended with GO in contents of 0%, 0.5%, 1%, 1.5%, 2%, and 2.5%. The permanent deformation behavior of the modified asphalt binders was evaluated based on the zero shear viscosity (ZSV) parameter through a steady shear test approach. Superpave fatigue test and the linear amplitude sweep (LAS) method were used to evaluate the fatigue behavior of the binders. A bending beam rheometer (BBR) test was conducted to evaluate the low-temperature cracking behavior. Furthermore, the storage stability of the binders was investigated using a separation test. The results of the ZSV test showed that GO considerably enhanced the steady shear viscosity and ZSV value, showing a significant contribution of the GO to the deformation resistance; moreover, GO modification changed the asphalt binder’s behavior from Newtonian to shear-thinning flow. A notable improvement in fatigue life was observed with the addition of GO to the binder based on the LAS test results and Superpave fatigue parameter. The BBR test results revealed that compared to the control asphalt, the GO-modified binders showed lower creep stiffness (S) and higher creep rate (m-value), indicating increased cracking resistance at low temperatures. Finally, the GO-modified asphalt binders exhibited good storage stability under high temperatures.

## 1. Introduction

In recent years, increasing traffic loading has led to the exploration of high-performance asphalt binders. Several studies showed that modification of base asphalt binders enhances their physical and rheological properties, resulting in minimizing common types of distresses, such as fatigue, low-temperature cracking, and rutting deformation, encountered in asphalt pavements [1,2,3,4]. Recently, nanomaterials, due to their high functional density, high specific surface area, and strong absorption, have demonstrated a great potential to improve the properties of the binder, leading to high performance and prolonged service life of the pavement [5,6]. Previous research indicated enhancement in the performance of asphalt binder by incorporating numerous kinds of nanomaterials, such as nano-silica, carbon nanotubes (CNT), and nanoclays, among others [7,8,9,10,11,12,13]. Ziari et al. [14] studied the physical and rheological properties of CNT incorporated asphalts. The findings demonstrated that CNT could substantially enhance the asphalt binders’ rutting and fatigue behavior and physical characteristics when applied at a relatively high dosage. Khattak et al. [15] reported a notable improvement in fatigue life and the visco-elastic response of asphalt binder by adding carbon nanofiber (CNF). Shafabakhsh et al. [16] found that employing nano-silica positively affected the base asphalt binder’s high-temperature performance and fatigue life. Filho et al. [17] showed that titanium dioxide (TiO_2_) nanoparticles exhibited a beneficial impact on the rheological properties, permanent deformation, and fatigue behavior of base asphalt binder (50/70) through different rheological approaches.

Because of its high specific surface area, layered structure, and excellent dispersion in aqueous solutions, graphene oxide (GO) has lately been considered a potential additive that can rapidly adsorb asphalt molecules, thus enhancing the performance characteristics of the binder and consequently of the asphalt mixture. GO is one of the nano-structured carbon materials reported to have good compatibility with asphalt due to its various surface oxygen-containing functional groups [18]. A few recent studies on asphalt modification using GO have been reported. Wu et al. and Zeng et al. [19,20], for example, investigated the use of GO as an anti-aging additive in binders. Their findings indicated that incorporating GO enhanced the binders’ resistance to oxidative and UV aging. Adnan et al. [21,22] found that the addition of GO improved the asphalt binder’s physical properties and enhanced the volumetric and strength properties of bituminous mixes.

Rutting is one of the most common types of failures in asphalt pavements, which is caused by repeated deformation due to high traffic loads and adverse weather conditions. Previous research found that using GO as an asphalt binder modifier improved the deformation resistance measured by the Superpave rutting parameter (G*/sinδ) and MSCR approach [18,23,24,25]. Several studies have shown that for modified binders, the G*/sinδ parameter is not very effective in evaluating the rutting behavior [26,27]. Zero shear viscosity (ZSV) is one of the advanced methods for assessing the asphalt binders’ rutting resistance that received significant attention from researchers recently [27,28,29]. The viscosity measured in shear deformation at a shear rate close to zero is known as the zero shear viscosity. ZSV has been identified as an indication of binder stiffness and permanent deformation performance under long-term loading [30]. The significance of the ZSV parameter has been validated through the rutting potential evaluation of bituminous mixtures [28,31]. The authors found no reported literature on using the ZSV approach to investigate the rutting behavior of GO incorporated asphalts. Therefore, the current research has attempted to use the ZSV approach to characterize the rutting deformation behavior of asphalt binders containing GO.

Another major problem in asphalt pavements is fatigue failure. The Superpave fatigue parameter (G*sinδ) was employed to investigate the fatigue behavior of asphalt binders incorporating GO [23]. However, this parameter does not capture actual damage to asphalt binder since the measurement is performed in the linear viscoelastic range [32]. New methods for fatigue evaluation, such as linear amplitude sweep (LAS), have gained considerable attention among researchers in recent years. LAS test evaluates the damage resistance of the binder using cyclic loading with linearly increasing load amplitudes. This approach applies the concept of viscoelastic continuum damage (VECD). The rate of damage accumulation shows the binder’s fatigue performance. The method has been validated by comparing mixture fatigue resistance and field performance [33]. There is limited research on assessing the fatigue behavior of GO-modified asphalt binders using effective methods, such as the LAS test. The current study employed the G*sinδ and LAS test, which was recently developed, to investigate the fatigue resistance of the GO-modified asphalt binders.

Concerning low-temperature cracking performance, the previous studies came to contradictory conclusions. Li et al. [23] reported enhancement in the low-temperature cracking resistance of the asphalt binder by applying GO based on the bending beam rheometer (BBR) analysis. Contrary to this, Liu et al. [18] concluded that the application of GO had no noticeable impact on the cracking behavior of the asphalt binders at low temperatures; in fact, the low-temperature cracking resistance of the binders was slightly reduced by adding certain GO contents. Hence, additional research in this regard is required to further understand the low-temperature cracking behavior of an asphalt binder containing GO.

As previously mentioned, so far, no study has been reported on GO-modified asphalt binder using ZSV. Likewise, limited research works are reported on the evaluation of fatigue performance of GO-modified binder using the advanced characterization approach. Although a couple of studies on low-temperature cracking performance are available, inconsistent outcomes have been reported. This work aimed to evaluate the deformation potential, fatigue, and low-temperature behavior of GO-modified binders. In this respect, the primary objectives were as follows:

- To investigate the impact of GO incorporation into the asphalt binder on the ZSV value through the steady shear test method;
- To investigate the impact of GO incorporation into the asphalt binder on fatigue performance using the Superpave parameter (G*sinδ) and LAS test approach;
- To evaluate the low-temperature cracking performance of GO-modified asphalt binders in terms of creep rate (m-value) and creep stiffness (S) using the bending beam rheometer testing;
- To investigate the impact of GO on the storage stability of asphalt binder using the separation test.

## 2. Materials and Methods

### 2.1. Materials

The base asphalt binder used in this study was a 60/70 penetration-grade asphalt binder. GO provided by Suzhou Tandem Graphene Technology CO., Ltd. (Suzhou, China) was used as an additive material. Table 1 presents the characteristic properties of the base binder and GO.

### 2.2. Sample Preparation

The modified asphalt binders were prepared using a high-shear mixing technique. First, the unmodified asphalt binder was heated to 160 °C in an oven and placed under the high-shear mixer at 4000 rpm. Certain amounts of GO (0.5%, 1%, 1.5%, 2%, and 2.5% GO by weight of the asphalt) were then gradually added into the asphalt while stirring for 45 min at 160 °C to obtain a homogenous mass.

### 2.3. Experimental Methods

#### 2.3.1. Steady Shear Test

**Figure 1:**
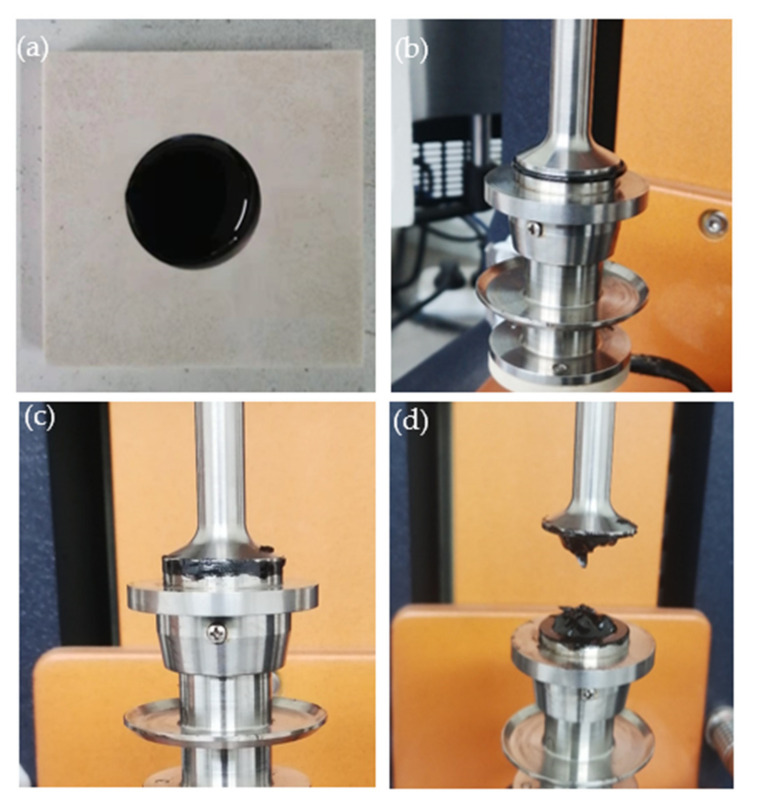
(**a**) test sample, (**b**) sample sandwiched between 25 mm-diameter parallel plates, (**c**) trimmed sample before loading, and (**d**) sample after the test.

The ZSV value of the GO binders was determined using a steady shear test. A DSR (DHR-2 model) instrument, shown in Figure 1, was used for this test, and the binder sample used had a diameter of 25 mm with a 1 mm thickness (shown in Figure 1a). Three decades of shear rates ranging from 0.01 to 10 s^−1^ were covered at 60 °C while the resulting change in viscosity was recorded. The ZSV, which serves as an indicator for the binder’s stiffness and permanent deformation resistance under long-term loading, was determined based on the Carreau model (Equation (1)) [39]. This model has been used to model the asphalt binder’s rheological (or shear-thinning) behavior [40,41].

(1) $$\eta =\eta_{\infty }+\frac{\eta_{0}-\eta_{\infty }}{{\left(1+{\left(\frac{\gamma }{\gamma_{c}}\right)}^{2}\right)}^{s}}$$

where η is the binder’s viscosity at various shear rates,$\;\eta_{0}$ is the zero shear viscosity, $\eta_{\infty }$ is infinite shear viscosity, $\gamma_{c}$ is the critical shear rate (where the shear-thinning begins), and *s* is the variable associated with the shear-thinning region’s slope.

This test also helped to understand the Newtonian and shear-thinning behavior of the binder by incorporating different GO contents. 

#### 2.3.2. Superpave Fatigue Test

**Figure 2:**
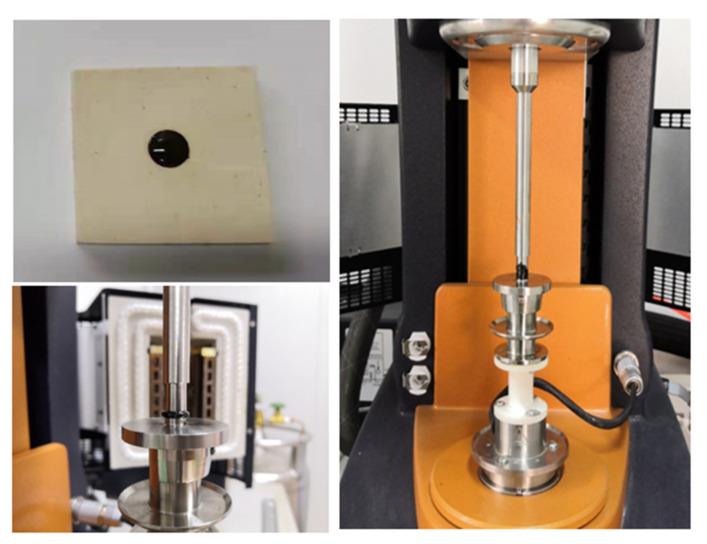
Fatigue test sample and set-up.

G*sinδ was used to evaluate the asphalt binders’ fatigue resistance according to the SHRP. The SHRP specified a maximum G*sinδ value of 5000 kPa. The test was carried out on PAV-aged samples because the asphalt binder becomes stiffer due to aging during its service life and becomes more susceptible to fatigue. The test was conducted using the DSR under shear loading at a frequency of 10 rad/s and 0.05% strain level. The samples used for the test had a diameter of 8 mm with a 2 mm thickness (shown in Figure 2). The complex modulus (G*) and phase angle (δ) were measured at 16 °C, 19 °C, and 22 °C, and fatigue parameter (G*sinδ) of the modified binders was calculated and compared.

#### 2.3.3. Linear Amplitude Sweep (LAS) Test

The LAS test was used to better understand the GO-modified binders’ fatigue behavior. The test was performed following AASHTO TP 101 [42] using DSR (model DHR-2, TA instruments, New Castle, DE, USA) on the PAV-aged modified binders at 25 °C and used 8 mm-diameter parallel plates with a 2 mm gap, as shown in Figure 2. First, a frequency sweep test with frequencies ranging from 0.2 to 30 Hz was applied at a strain level of 0.1% for evaluating the undamaged material. The amplitude sweep test (0.1–30% strain level) was then performed at a frequency of 10 Hz. The binder samples’ fatigue life was calculated using the continuum damage approach.

First, the log of storage modulus was plotted against the log of applied frequency to obtain a straight line from the frequency sweep results. The slope (m) is obtained, and the parameter α is calculated as the inverse of the m-value. The following equation can then be applied to calculate the accumulated damage in the sample.

(2) $$D\left(t\right)≅\sum_{i=1}^{N}{\left[\pi \gamma_{0}^{2}(C_{i-1}-C_{i}\right]}^{\alpha /\left(1+\alpha \right)}{\left(t_{i}-t_{i-1}\right)}^{1/\left(1+\alpha \right)}$$

where *D(t)* is the accumulated damage, *γ_o_* = percent of applied strain (%), and *C_i_* = G*(t)/G* (initial), *t* = testing time, sec, and G* = complex modulus.

The following power law can be used to fit the relationship between the integrity parameter ($C_{\left(t\right)}$) and damage accumulation:

(3) $$C_{\left(t\right)}=C_{0}-C_{1}{\left(D\right)}^{C_{2}}$$

where *C*_0_, *C*_1,_ and *C*_2_ are parameters of the curve-fitting. Equation (4) was further used to calculate the damage at the point of failure at the peak shear stress.

(4) $$D_{f}={\left(\frac{C_{0}-C_{\mathrm{at}\;\mathrm{peak}\;\mathrm{stress}}}{C_{1}}\right)}^{1/C_{2}}$$

The binders’ fatigue life ($N_{f}$) can be calculated using the following equation:

(5) $$N_{f}=A\times {\left(\gamma_{max}\right)}^{-B}$$

where *A* and *B* are regression coefficients and $\gamma_{max}$ is the maximum sample strain (%).

The regression parameters can be obtained as follows:

(6) $$A=\frac{f{\left(D_{f}\right)}^{1+\left(1-C_{2}\right)\alpha }}{\left(1+\left(1-C_{2}\right)\alpha \right)\times \left(\pi C_{1}C_{2}\right)\alpha }$$

(7) B=2α

where k = 1 + (1 − *C_2_*) α and *f* = loading frequency (10 Hz).

#### 2.3.4. Bending Beam Rheometer (BBR)

BBR test was employed to assess the low-temperature cracking behavior of the asphalt binder following AASHTO T313-12 [43]. The test’s main outputs are creep stiffness and creep rate (m-value). These parameters show the ability of an asphalt binder to resist cracking at low temperatures. The test was performed on PAV-aged binders at −6, −12, −18, and −24 °C. Rectangular binder beam samples with dimensions of 125 × 12.5 × 6.35 mm^3^ were used for this purpose. The samples were conditioned in an ethanol bath at the required test temperature for 60 min before testing. Each sample was subjected to a constant load of 0.98 N for 240 s. The resulting midpoint deflection at loading times of 8, 15, 30, 60, 120, and 240 s was measured and plotted against time. The creep rate (m-values) and creep stiffness (S) of the asphalt binders at 60 s were calculated and analyzed.

#### 2.3.5. Storage Stability Test

The phase separation test was used to investigate the effect of GO on asphalt binder’s high-temperature storage stability. The samples were poured into an aluminum tube 140 mm in height and 25 mm in diameter and placed in an oven for 48 h at 163 °C. After that they were taken out of the oven and refrigerated for 4 h. Finally, the tubes were cut to give three equal pieces, and a sample was taken from the bottom and top sections of the tube to perform a softening point test. The differences in softening points between the top and bottom pieces were determined to assess the modified binders’ resistance to phase separation.

## 3. Results and Discussion

### 3.1. Zero Shear Viscosity

**Figure 3:**
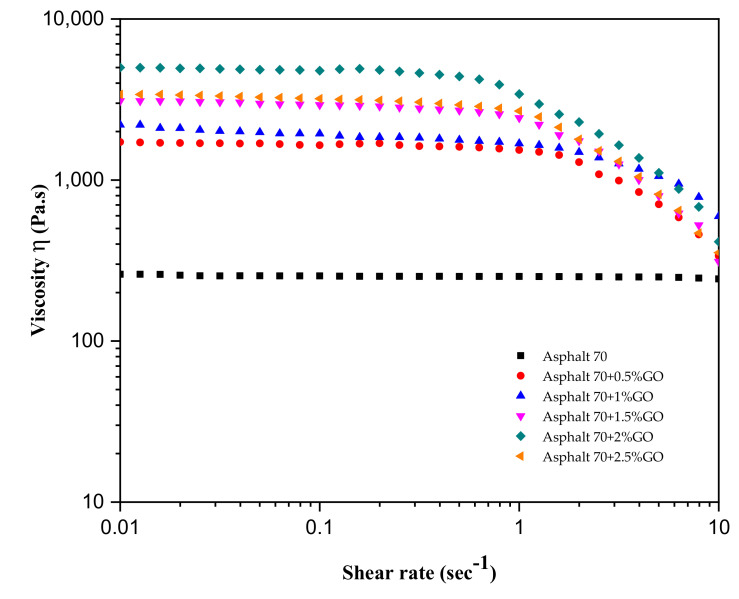
Viscosity versus shear rate at 60 °C for different GO-modified binders.

The impact of varying shear rates on the viscosity of the base and GO-modified asphalt binders at 60 °C is illustrated in Figure 3. As shown, the unmodified asphalt demonstrated Newtonian behavior up to the applied shear rate of 9 s^−1^. The viscosity remained constant regardless of the applied shear rate. The incorporation of GO greatly enhanced the binder’s viscosity at the tested temperature; moreover, the GO binders exhibited a change in behavior from Newtonian to non-Newtonian (shear-thinning flow) within a shear rate range of 0.1 to 1 s^−1^. The shear rate values at the transition region for the 0.5%, 1%, 1.5%, 2% and 2.5 GO-modified samples were about 1.69, 1.57, 0.9, 0.51, and 0.77 s^−1^, respectively. This demonstrates that as GO content increased up to 2%, the Newtonian region decreased, and the shear-thinning response increased. The Newtonian region slightly increased with a higher GO content (2.5%).

**Figure 4:**
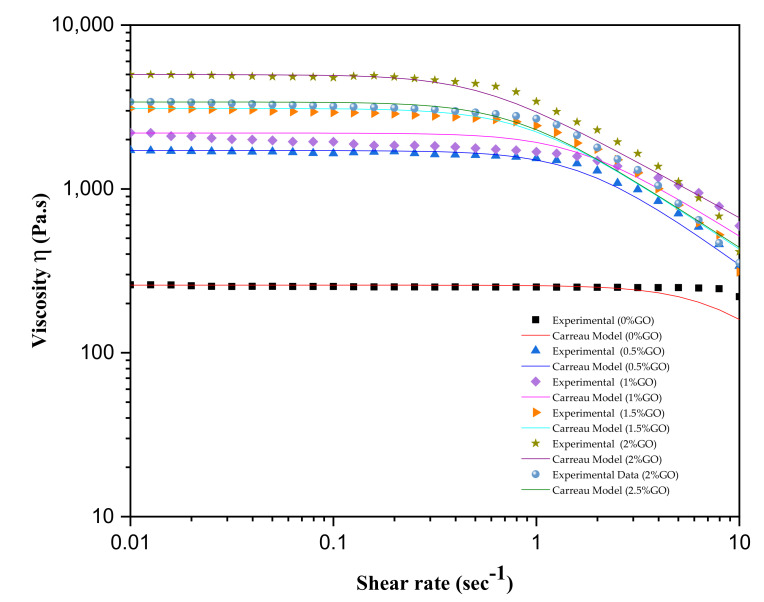
Carreau model fitted curves for GO-modified binders.

The Carreau model fitted the experimental results very well for GO-modified asphalt binders, as shown in Figure 4. Table 2 presents the ZSV value and different Carreau model parameters. The ZSV has been proposed as an essential factor for assessing the rutting behavior of the asphalt binder. As shown in Table 2, the ZSV value was considerably enhanced by applying GO. The increase in ZSV could be attributed to the improved stiffness caused by GO modification [21]. Owing to its surface chemical functionalities and the high specific area, GO can easily blend with asphalt molecules and form a strong interfacial bond, therefore enhancing the cohesion and stiffness and positively impacting the control asphalt’s deformation resistance [18]. The highest ZSV value was indicated with the 2% GO binder. This shows that the asphalt’s hardness was enhanced by applying GO to a certain extent. Excess content (or 2.5%) possibly reduces GO material’s homogeneity, decreasing the entire composite’s stiffness and thus the ZSV of the binder. According to our previous study of the asphalt binder, the 2% GO had the most significant improvement in stiffness of all the GO binders [21]. Overall, the rutting deformation resistance of the control asphalt was enhanced with the incorporation of GO, as indicated by the increase in the ZSV values. Previous literature reported similar findings for acid-treated fly ash incorporated asphalt [40] and nano-silica and CNT modified binders [8,41].

### 3.2. Superpave Fatigue Parameter

**Figure 5:**
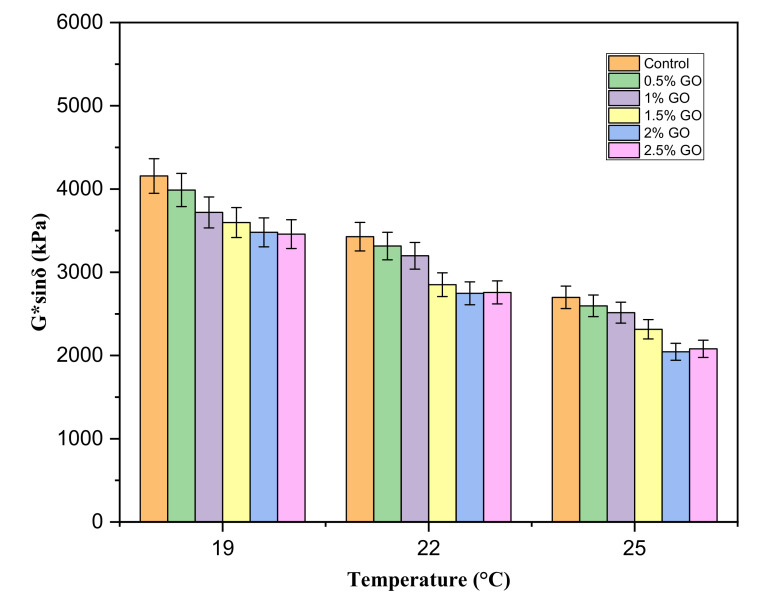
G*sinδ values of GO-modified binders at 19 °C, 22 °C, and 25 °C.

Figure 5 shows the fatigue performance (G*sinδ values) of the control and GO-modified binders based on Superpave fatigue test at 16 °C, 19 °C, and 22 °C. A lower value of G*sinδ implies better fatigue resistance. Compared with the control asphalt, the GO-modified binders presented lower G*sinδ values (shown in Figure 5). This indicates that GO can enhance the fatigue resistance of the control asphalt binder (70#). Since the G*sinδ values were obtained below the binders’ maximum limit of 5000 kPa for PAV-aged asphalt binders, the GO-modified binders’ fatigue resistance at intermediate temperatures was found to be adequate. The presence of GO in the binder improved the asphalt’s stiffness due to the strong bonding between the binder and GO and can effectively retard the aging process by delaying the heat loss [10]. Based on this outcome under the tested conditions for fatigue at different temperatures, it can be concluded that by incorporating GO, fatigue cracks caused by asphalt will not occur in the pavement when subjected to repeated loads.

### 3.3. Linear Amplitude Sweep Test

**Figure 6:**
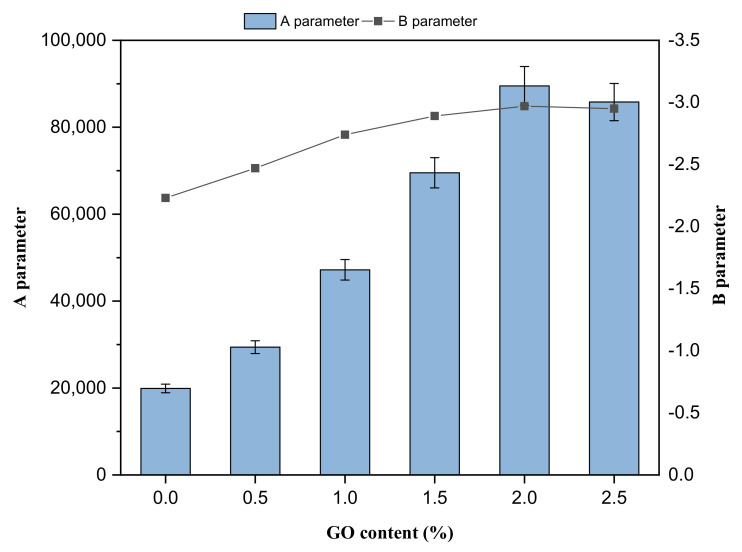
The A and B parameters of binders from linear amplitude sweep tests.

Figure 6 shows the A and B parameters for the investigated asphalt binders obtained from the LAS test and based on VECD analysis. The variation in the material’s integrity caused by accumulated damage is represented by parameter A. The material’s integrity should be maintained throughout the cycles, as measured by the loss modulus. A higher value of the A parameter implies the binder maintains its initial integrity. As shown in this figure, the GO-modified binders’ A parameter values were higher than those of the control asphalt binder. On the other hand, the B parameter is related to the asphalt binder’s sensitivity to an increase in the level of deformation. It is associated with the slope of the straight line of frequency vs. storage modulus. As shown in Figure 6, the unmodified asphalt binder showed the highest B parameter value compared to the modified samples. A decreasing trend in the B value was observed with increased GO content.

**Figure 7:**
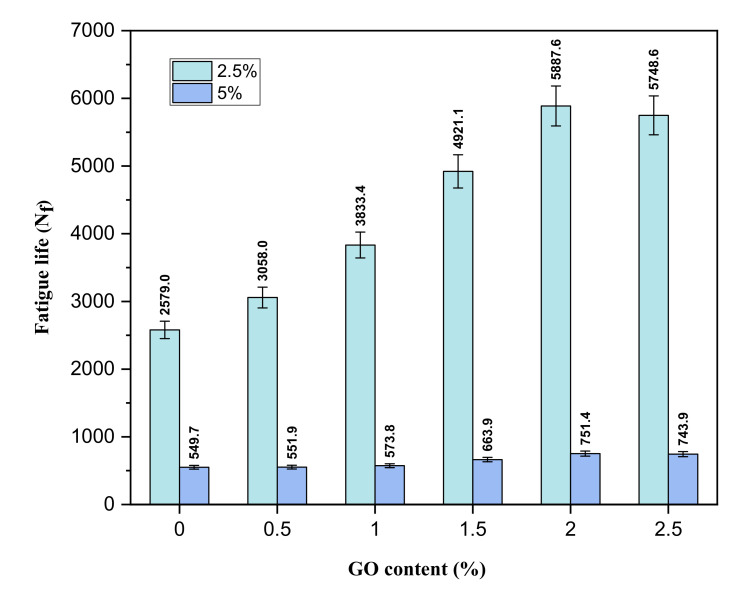
The fatigue life of GO-modified binders from the LAS test at strain levels of 2.5% and 5%.

The fatigue life of GO incorporated asphalts at 2.5% and 5% strain levels are depicted in Figure 7. The addition of GO improved the asphalt binder’s fatigue life (*N_f_*) significantly. The 2% GO-modified binder demonstrated a higher number of failure cycles compared to other modified samples, while the control sample exhibited the lowest fatigue life at both strain levels. The improvement in fatigue resistance can be attributed to the large surface area of GO, leading to strong bonding between the GO and binder, therefore preventing the growth of micro-cracks and, subsequently, enhancing the asphalt’s fatigue resistance; also, the softening effect of GO on asphalt due to a slight amount of CO_2_ could contribute to the enhancement. The addition of 2.5% GO resulted in agglomerates of GO in the matrix. Thus, leading to a low softening effect in the binder and decreasing the fatigue life. Based on this result, it can be concluded that incorporating GO in the asphalt binder can improve its fatigue resistance. A similar finding has been reported by Singh et al. [24].

### 3.4. BBR Test

**Figure 8:**
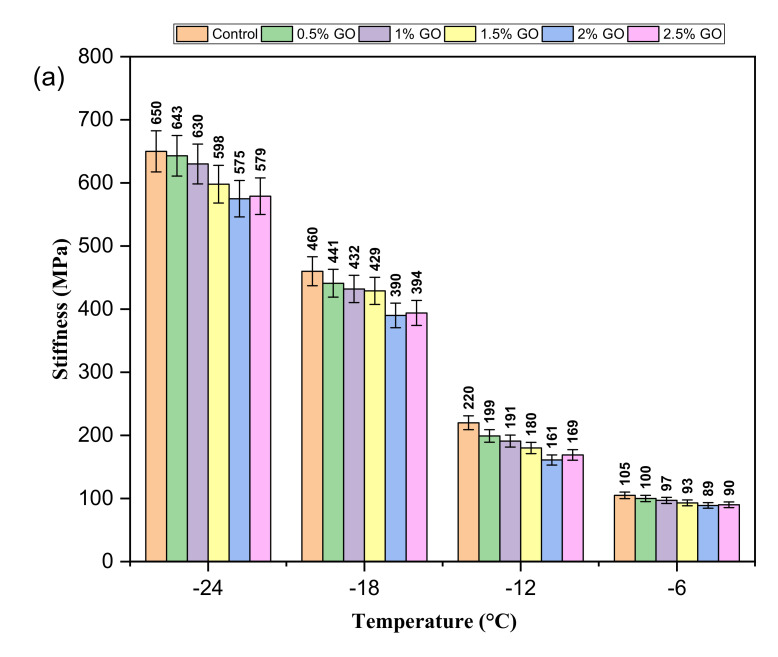

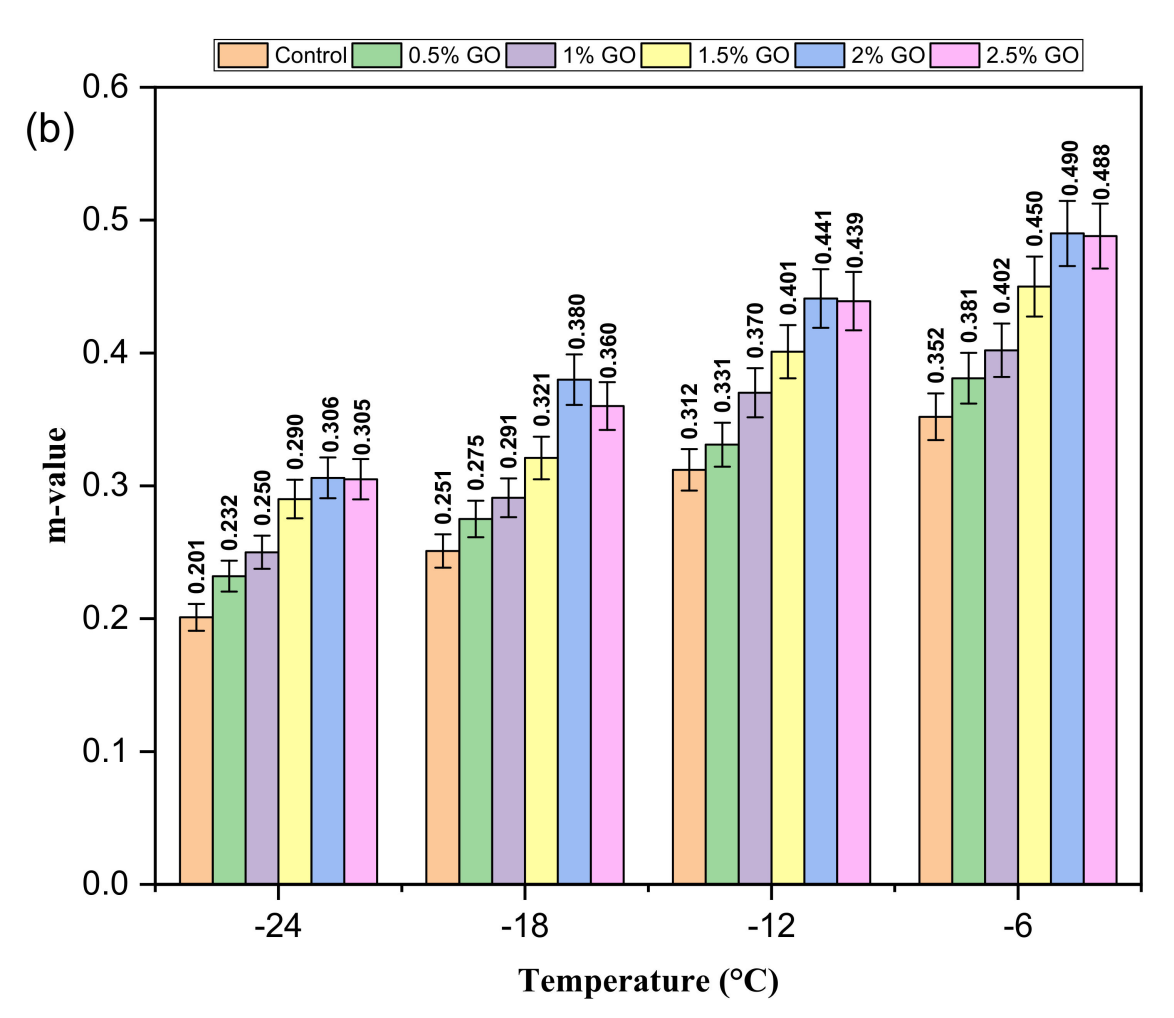
BBR test results: (**a**) creep stiffness (S) and (**b**) creep rate (m-value) of GO-modified binders at different temperatures.

To evaluate the GO-modified asphalt binders’ low-temperature performance, a BBR test was performed at temperatures of −24, −18, −12, and −6 °C. The creep stiffness (S) and creep rate (m-value) were obtained, which are the criteria used for assessing asphalt’s low-temperature cracking resistance. A lower S-value and a higher m-value would indicate improved anti-cracking performance at low-temperature. Figure 8 shows the BBR test results of the modified binders. The dependency of the asphalt’s low-temperature properties on the testing temperature can be seen in these figures. As shown in Figure 8a, the S-value of the control binder was lower than that of the GO-modified binders regardless of the test temperature. This indicates that the low-temperature flexibility of the asphalt was enhanced by incorporating GO. The trend decreased until 2% GO and increased at 2.5% GO. Meanwhile, as shown in Figure 8b, increasing trends in m-value were observed with increased GO content up to 2% and then slightly reduced at 2.5% GO. This could be attributed to the decrease in the softening effect of GO as a result of agglomerates of GO in the matrix by adding 2.5% [23]. The finding suggested that the addition of GO will help in enhancing the ability of the binder to relax stresses. AASHTO M 320 specified a maximum S-value of 300 MPa and a minimum m-value of 0.300. At −12 °C and −6 °C, all the asphalt binders satisfied the criteria mentioned above. While at −18 °C, corresponding to the actual field temperature of −28 °C, the binders modified with 0% (control), 0.5%, and 1% GO did not meet the stiffness and m-value requirements.

Based on these results, it can be deduced that GO reduced the cracking potential of the asphalt binder and has a beneficial impact on resistance to cracking. A similar enhancement in low-temperature cracking performance with the incorporation of GO was observed by Li et al. [23].

### 3.5. Storage Stability

The storage stability test determines whether modified binders have an appropriate level of phase separation resistance when stored at high temperatures. It is required that a modified binder must have a softening point difference (SPD) of less than 2.2 °C in order to perform satisfactorily. Table 3 shows the SPD values of the GO-modified asphalt binders. As seen, the SPD values of the modified binders are within the acceptable limit (<2.2 °C). This shows that although stored at high temperature, the mix of GO and binder were very well dispersed and remain in a homogenous and stable condition; therefore, incorporating GO up to 2.5% in the control binder (asphalt 70) may not result in phase separation during high-temperature storage. The uniform dispersion GO in the binder could be attributed to the GO’s large surface area and surface chemical functionalities (like carboxylic, hydroxyl, carbonyl, and epoxy), making it easy to blend with asphalt molecules and form interfacial forces.

## 4. Conclusions

This work examined the impact of GO on asphalt binder properties. The deformation behavior of GO incorporated binders was explored by employing the ZSV approach. The fatigue life was characterized using the Superpave fatigue factor and linear amplitude test. Low-temperature cracking resistance was investigated by the method of BBR. In addition to the rheological characterization, a phase separation test was conducted to assess the binders’ storage stability. Based on the findings of this investigation, the following conclusions can be drawn:

- GO modification increased the steady shear viscosity and the shear-thinning behavior and reduced the Newtonian plateau zone of the asphalt binder. Moreover, the ZSV values of the GO-modified binders were higher than that of the control asphalt binder, indicating that GO can increase the resistance of the asphalt to rutting deformation.
- The significant improvement in Superpave fatigue parameter (G*sinδ) and LAS fatigue life (N_f_) revealed that adding GO into the asphalt binder can enhance the fatigue performance.
- The results of the BBR test showed that the incorporation of GO increased the relaxation parameter (m-value) and decreased the creep stiffness, indicating a significant contribution to thermal cracking resistance at low temperatures.
- The results suggested that 2% GO was the optimal content for improving the binder performance.
- Storage stability analysis revealed that GO-modified binders would resist phase separation when stored at high-temperature.

## Figures and Tables

**Table 1:** Characteristic properties of the base binder and GO.

Material	Property	Value	Requirement	Method
Base binder	Penetration at 25 °C (0.1 mm)	68.5	60–70	ASTM D5 [34]
	Softening point (°C)	49	49 (Min)	ASTM D36 [35]
	Viscosity at 135 °C (Pa s)	0.55	<3 Pa s	ASTM D4402 [36]
	Ductility at 25 °C (cm)	138	100 (Min)	ASTM D113 [37]
	Rutting factor, G*/sin δ (Pa) at 64 °C	1649.7	>1 kPa	ASTM D7175 [38]
GO	Purity (%)	>95	–	–
	Layer	1–5	–	–
	Thickness (nm)	1.0–1.77	–	–
	Diameter (µm)	10–50	–	–
	SSA (m^2^/g)	300–450	–	–

**Table 2:** Carreau model parameters for GO-modified binders.

Binder Type	$\mathrm{ZSV},\;\eta_{0}\;(\mathrm{Pa}.s)$	$\gamma_{c}\;(s^{-1})$	s	Regression Coefficient, R^2^
Asphalt 70	257.7	9	0.60	0.997
Asphalt 70 + 0.5% GO	1715.6	1.7	0.45	0.993
Asphalt 70 + 1% GO	2194.1	1.58	0.39	0.982
Asphalt 70 + 1.5% GO	3097.4	0.9	0.41	0.994
Asphalt 70 + 2% GO	4984.6	0.52	0.34	0.996
Asphalt 70 + 2.5% GO	3392.2	0.78	0.40	0.980

**Table 3:** Storage stability (separation test results) of GO-modified binders.

Binder Type	Softening Point (°C)	Softening Point Top (°C)	Softening Point Bottom (°C)	SPD (°C)
Asphalt 70 + 0.5% GO	49.4	50.8	50.4	0.4
Asphalt 70 + 1% GO	50.1	51.9	51.6	0.3
Asphalt 70 + 1.5% GO	51.9	53.0	52.8	0.2
Asphalt 70 + 2% GO	53.0	54.4	54.6	0.2
Asphalt 70 + 2.5% GO	51.4	52.9	52.6	0.3

## Data Availability

Did not report any data.
